# Rituximab for Treatment of Membranoproliferative Glomerulonephritis and C3 Glomerulopathies

**DOI:** 10.1155/2017/2180508

**Published:** 2017-05-09

**Authors:** Michael Rudnicki

**Affiliations:** Department of Internal Medicine IV–Nephrology and Hypertension, Medical University Innsbruck, Anichstrasse 35, 6020 Innsbruck, Austria

## Abstract

Membranoproliferative glomerulonephritis (MPGN) is a histological pattern of injury resulting from predominantly subendothelial and mesangial deposition of immunoglobulins or complement factors with subsequent inflammation and proliferation particularly of the glomerular basement membrane. Recent classification of MPGN is based on pathogenesis dividing MPGN into immunoglobulin-associated MPGN and complement-mediated C3 glomerulonephritis (C3GN) and dense deposit disease (DDD). Current guidelines suggest treatment with steroids, cytotoxic agents with or without plasmapheresis only for subjects with progressive disease, that is, nephrotic range proteinuria and decline of renal function. Rituximab, a chimeric B-cell depleting anti-CD20 antibody, has emerged in the last decade as a treatment option for patients with primary glomerular diseases such as minimal change disease, focal-segmental glomerulosclerosis, or idiopathic membranous nephropathy. However, data on the use of rituximab in MPGN, C3GN, and DDD are limited to case reports and retrospective case series. Patients with immunoglobulin-associated and idiopathic MPGN who were treated with rituximab showed partial and complete responses in the majorities of cases. However, rituximab was not effective in few cases of C3GN and DDD. Despite promising results in immunoglobulin-associated and idiopathic MPGN, current evidence on this treatment remains weak, and controlled and prospective data are urgently needed.

## 1. A Brief Introduction on Membranoproliferative Glomerulonephritis

From a traditional perspective membranoproliferative glomerulonephritis (MPGN) has been defined by a morphological pattern of glomerular injury, in which electron-dense immunoglobulins and/or complement components are deposited between endothelial cells and the basement membrane [1]. Increased matrix deposition and hypercellularity lead to typical changes seen by light microscopy: thickening of the capillary wall often appears as a double contour (“tram track,” “membrano-”) and mesangial cells interpose in the newly formed second layer of basement membrane (“proliferative”). Based on these morphological features MPGN has historically been classified into three types: in the most frequent MPGN type 1 light microscopy typically shows double contours of the capillary walls and mesangial proliferation, and electron microscopy reveals subendothelial electron-dense deposits [2]. These deposits can be positive for either immunoglobulins or complement factor C3, or for both. On electron microscopy MPGN type 2 reveals a distinctive feature of extremely electron-dense material deposited throughout the whole basement membrane, which on immunohistology stains positive for C3 but usually not or only sparsely for immunoglobulins [3]. A typical feature of MPGN type 3 is the presence of subepithelial immune deposits in addition to subendothelial and mesangial immune deposits, which can be positive for immunoglobulins or C3 like in type 1 MPGN [4].

In the last decade novel insights in pathogenic mechanisms have changed our understanding and the classification of MPGN [5]. It has been increasingly recognized that in some cases the deposition of immunoglobulins in the first place is followed by a secondary complement activation (i.e., “immunoglobulin-associated MPGN”). Immunoglobulin-associated or immune-complex mediated MPGN is often secondary to infections (viral such as hepatitis B or C; bacterial such as endocarditis, atrioventricular shunts, visceral abscesses, mycoplasma, or protozoal such as malaria or schistosomiasis), can be caused by cryoglobulinemia (with or without hepatitis B or C), or represents a poststreptococcal glomerulonephritis. Other causes are autoimmune diseases including systemic lupus erythematosus, or malignancies such as lymphoproliferative disorders including monoclonal IgG gammopathies. If no obvious cause can be identified then the case is termed idiopathic MPGN, although some authors argue that such rare cases might represent a C3 glomerulonephritis with some immunoglobulin deposits, and therefore an underlying pathology of the complement system has to be excluded.

In other cases a primary pathology of complement control results in the deposition of C3 without a significant deposition of immunoglobulins (i.e., C3 glomerulonephritis-C3GN, or dense deposit disease, DDD). The difference between DDD and C3GN is represented by the fact that DDD is characterized by extremely electron-dense deposits in the glomerular basement membrane, while the glomerular changes of C3GN are more heterogenous. Although the most frequent histologic pattern identified by light microscopy is MPGN other glomerular changes such as mesangial proliferative glomerulonephritis and endocapillary proliferative GN with or without crescents have also been described in patients with DDD [6].

However, both pathologies are a consequence of abnormal glomerular accumulation of C3 due to acquired or genetic disorders of complement regulation [7]. Hyperactivation of the alternative pathway of complement as seen in C3GN and DDD can be associated with the presence of C3 nephritic factors (C3Nefs), which stabilize C3 convertase or its components against complement factor H- (CFH-) mediated decay, thus leading to prolonged and dysregulated activation of the complement system. Although C3Nefs are found in 40–80% of patients with C3GN/DDD their correlation with disease course and outcome has been questioned [8]. Antibodies against other components of the complement system have also been identified, such as anti-CFH, anti-complement factor B (CFB), anti-C4, or anti-C3b [9].

Multiple genetic causes have been identified for C3GN and DDD. These include loss of function mutations of CFH and CFB, or gain of function mutations of C3. Furthermore, mutations of the CFHR5 gene or copy number variations of the CFHR gene cluster have been reported [10].

## 2. Clinicopathological Features and Treatment of MPGN

Clinical presentation of patients with MPGN may be highly variable and similar to that in other types of glomerulonephritis. Patients can present with microscopic hematuria with or without mild proteinuria to nephrotic range proteinuria or even full blown nephrotic syndrome with or without hypertension and renal function decline. Renal prognosis is usually determined by the degree of proteinuria and reduction of eGFR at time of presentation and during follow-up, like in most proteinuric renal diseases [11]. Another important adverse prognostic factor is the degree of tubulointerstitial fibrosis on renal biopsy rather than the disease type or severity of glomerular changes [12].

There are several issues which have to be taken into account when treating patients with MPGN, C3GN, or DDD. In immunoglobulin-associated MPGN at least partial resolution of MPGN occurs when the primary cause is successfully treated, for example, antiviral therapy in hepatitis B or C [13], antimicrobial therapy in infectious diseases [14], or chemotherapy in chronic lymphocytic leukemia [15] and multiple myeloma [16]. In the case of MPGN secondary to a monoclonal gammopathy the term MGRS (monoclonal gammopathy of renal significance) should be used [17]. Although the optimal treatment in these cases is uncertain, some authors suggest treating this condition like multiple myeloma [18].

In patients with a HCV-related renal disease (presumably MPGN) and cryoglobulinemia with nephrotic proteinuria and evidence of progressive kidney disease, treatment with plasmapheresis, rituximab, or cyclophosphamide in conjunction with steroids and antiviral therapy may be most useful, but data are limited and treatment approaches have to be individualized [19].

There are no randomized controlled trials upon which treatment decisions for idiopathic MPGN can be based, once secondary causes have been excluded. Patients with nonnephrotic proteinuria and stable renal function may be treated with supportive measures such as renin-angiotensin-aldosterone (RAS) blockade and consequent blood pressure control alone, since these patients have a favourable long term renal outcome [20]. According to recent KDIGO glomerulonephritis guidelines immunosuppressive treatment should only be started in those patients with nephrotic range proteinuria and renal function decline, and also in this setting quality of evidence is poor [21]. Furthermore, in most and particularly in the early reports patients were classified by light and electron microscopy into MPGN type 1 and type 3, while patients with type 2 were often discussed separately. Since classification has changed in recent years, the results of these case reports and case series have to be interpreted with caution.

The efficacy of glucocorticoids has been tested in 80 children with MPGN, nephrotic range proteinuria, and preserved renal function. Although therapy with 40 mg/m^2^ prednisone up to 41 months showed a lower rate of treatment failure and a borderline significant stabilization of renal function at 10 years of follow-up, steroid therapy was associated with substantial toxicity [22]. Data on the efficacy of cyclophosphamide are conflicting. Cattran et al. did not show any difference in patient survival, renal function decline, and proteinuria at 2 years as compared to a control group [23]. On the contrary the rate of complete remission after 10 months was 79% in an (uncontrolled) observational study of 19 patients by Faedda et al. [24]. Only limited data from small case series are available for mycophenolate mofetil [25], cyclosporine A [26], and tacrolimus [27] for treatment of immunoglobulin-associated MPGN. Both C3GN and DDD are extremely rare diseases and data on treatment are even more limited than for immunoglobulin-associated or idiopathic MPGN. On the level of case reports therapies including plasma infusion, plasmapheresis, glucocorticoids, cyclophosphamide, mycophenolate mofetil, eculizumab, or calcineurin inhibitors have been reported with varying degrees of efficacy [28]. In summary, the overall benefit of standard immunosuppressive therapy in the setting of immunoglobulin-associated MPGN, C3GN, and DDD might be very limited and quality of evidence is very weak. Therefore, the recent KDIGO glomerulonephritis guidelines state that progressive renal function decline remains the only indication for (intensive) immunosuppressive treatment [21].

## 3. Rituximab in Immunoglobulin-Associated MPGN

Rituximab is a chimeric mouse/human monoclonal antibody targeting the CD20 surface antigen on B-lymphocytes, selectively depleting these cells. It has to be noted that CD20 is expressed on most types of B-lymphocytes (e.g., pre-B-cells, immature B-cells, naïve B-cells, germinal-center B-cells, and memory B-cells) but not on pro-B-cells, plasmablasts, and plasmacells [29]. Furthermore, rituximab seems to have a direct protective effect on podocytes. It has been shown that rituximab regulates the sphingomyelin phosphodiesterase acid-like 3b protein and acid sphingomyelinase activity, thus stabilizing the actin cytoskeleton and preventing apoptosis of podocytes [30]. Since in immunoglobulin-associated MPGN the deposition of immunoglobulins is probably the primary event and complement activation as well as unspecific glomerular and tubular changes are a consequence thereof, depletion of B-cell autoantibody production by rituximab represents a reasonable approach.

In fact, treatment with rituximab has been effective in MPGN caused by chronic lymphocytic leukemia, but it is unclear if the beneficial effect was due to its immunosuppressive characteristics (i.e., antibody depletion) or primarily due to an effect on leukemic cells [15, 31, 32]. Also in MPGN associated with mixed cryoglobulinemia with or without HCV infection rituximab has been shown to be effective in conjunction with glucocorticoids, although severe adverse effects such as fatal infections have been reported [33–35].

Since truly idiopathic MPGN can only be established after exclusion of secondary causes such as mentioned above, its prevalence is decreasing. Nevertheless, some reports have been published on the efficacy and safety of rituximab in this setting (Table 1). In an open-label prospective trial in 6 patients with type 1 MPGN (4 idiopathic and 2 with cryoglobulinemia) who did not receive any immunosuppressants prior to the study 1000 mg rituximab was administered on day 1 and day 15 and outcome was change in proteinuria [36]. These patients had slightly reduced renal function with a creatinine clearance of 48 ± 13 ml/min/1.73 m^2^. B-cells were effectively depleted and 24 h proteinuria was significantly reduced from 3.9 ± 2.0 g to 2.1 ± 2.3 g after 12 months. Interestingly those 2 patients with cryoglobulinemia showed the best response with complete remission after 12 months. Renal function remained stable in all 6 patients and no adverse events were noted.

In two reports on treatment of subjects with primary glomerular diseases with rituximab MPGN patients were included. In a retrospective report of 24 patients with various primary glomerulopathies one patient had MPGN. Treatment consisted of a single dose of rituximab of 375 mg/m^2^ (maximum of 500 mg), which was associated with a complete B-cell depletion. Urinary protein excretion decreased from 9.8 g/day to 1.8 g/day after 6 months [37]. In another single-center retrospective case review of 24 patients with primary glomerulonephritides 2 patients with MPGN were included. One patient achieved a complete and the other a partial remission after one or two doses of rituximab (375 mg/m^2^). Interestingly one of these patients presented with a crescentic rapid-progressive glomerulonephritis (RPGN) and progressed to dialysis after treatment with rituximab. However, 5 months later he was able to discontinue dialysis and was even in complete remission after 14 months [38].

In summary, rituximab seems to be effective in immunoglobulin-associated MPGN caused by lymphoproliferative disorders, in cryoglobulinemia with or without viral infections, or in truly idiopathic MPGN. However, the level of evidence is extremely low for all of these indications and caution is warranted due to several limitations: (1) the effect of various concomitant immunosuppressive medications such as glucocorticoids has not been evaluated; (2) a comparison with plasmapheresis, cyclophosphamide, or any other intensive immunosuppressive protocol is lacking, particularly in cryoglobulinemia; (3) the effect of modern anti-HCV therapy on MPGN (with or without cryoglobulinemia) has not been studied so far, but a favourable effect and an improvement of the safety profile of a concomitant therapy with rituximab and other immunosuppressants seems likely; (4) the regimen of rituximab is not clear and it is also not clear if rituximab should be repeated upon B-cell repopulation.

## 4. Rituximab in C3GN and DDD

In contrast to immunoglobulin-associated MPGN the primary pathology of C3GN and DDD is an excessive activation of the alternative complement pathway with glomerular deposition of C3 without a significant deposition of immunoglobulins. It may be hypothesized that in the presence of autoantibodies such as C3Nefs, which lead to uncontrolled activation of the complement cascade and finally in end-organ damage, a B-cell depletion with rituximab may be effective [39]. However, there are few case reports published on the use of B-cell depleting therapy to decrease production of C3Nef and subsequently to reduce proteinuria and stabilize or even improve renal function (Table 2). In one report a 34-year-old patient with DDD was treated solely with rituximab [40]. Low C3 and normal C4 indicated an activation of the alternative complement pathway, and C3Nef was found to be positive. CFH and complement factor I (CFI) were normal, and anti-CFH was negative. Serum creatinine was 235 *μ*mol/L. After initial treatment with ACE-inhibitors and good blood pressure control, the patient remained nephrotic and received 4 weekly doses of 700 mg rituximab as the only immunosuppressive therapy. Within 6 months a complete remission of the nephrotic syndrome was achieved, which lasted until 18 months. At this timepoint B-cell counts began to rise and the initial course of rituximab was repeated. After 30 months of follow-up the patient had a stable renal function and proteinuria remained < 0.5 g/day. Remarkably, C3Nef remained positive throughout the study, and C3 levels were always low, which questions the pathogenetic role of C3Nef/the complement system, at least in this patient.

In an 11-year-old girl with DDD, nephrotic syndrome, normal renal function, C3Nef positivity, and low C3 a single dose of 375 mg/m^2^ rituximab was administered which resulted in complete B-cell depletion [41]. After 5 months proteinuria did not resolve and serum creatinine increased. Genetic analysis revealed factor H variants which have previously been associated with DDD, and the terminal complement complex showed a high level of activity, further supporting the hypothesis of activation of the alternative complement pathway. Finally, a therapy with eculizumab over 48 weeks resulted in stabilization of renal function and partial remission of proteinuria.

In a similar report an 8-year-old boy was diagnosed with DDD with signs of activation of the alternative complement pathway (low C3, normal C4, genetics of CFH, CFI and MCP normal, and anti-CFH negative) and positivity of C3Nef [42]. Initial treatment with RAS-blockade, steroids and MMF resulted in complete remission, and after 11 months steroids were withdrawn. Four months later a relapse of the nephrotic syndrome occurred which did not respond to steroid reinitiation, and 2 doses of rituximab (375 mg/m^2^) were given. However, this therapy resulted in acute renal failure which required dialysis treatment. Renal biopsy revealed active DDD with extracapillary crescents which underlined the ineffectiveness of rituximab in this patient. Finally, treatment with eculizumab resulted in a quick and complete response.

A very similar case was reported by Payette et al. [43]: a 5-year-old boy with a C3GN was treated unsuccessfully with steroids, MMF, and also rituximab but responded quickly to a therapy with eculizumab with a decrease of proteinuria from 5.3 g/day to 1.7 g/day. In addition to a positive C3Nef and low C3, also anti-CFH was found to be positive. Interestingly, anti-CFH was reduced by rituximab without any effect on proteinuria.

In summary, the available evidence from single case reports does not support rituximab as an effective treatment for patients with C3GN or DDD, and the role of C3Nefs as a potential target for B-cell depleting therapy remains to be further elucidated. It has to emphasized that the activity of C3Nefs varies during the course of disease without any association to clinical presentation or treatment. Further, C3Nefs are heterogenous and detection might be challenging, and finally C3Nefs have also been found in other renal diseases [44]. In the majority of cases the underlying pathology of C3GN and DDD is an excessive activation of the alternative complement pathway. Therefore, it is reasonable that treatment with eculizumab, a humanized monoclonal antibody that binds to C5 and inhibits activation of the terminal complement complex, may provide a more targeted therapy than rituximab for patients with these diseases. However, complement inhibition in C3GN and DDD does not translate into clinical improvement in all patients, but again quality of evidence is limited. Bomback et al. reported on 6 adult patients with either DDD or C3GN (including 2 with recurrence of the disease after kidney transplantation) treated with eculizumab for 12 months [45]. Two patients showed improvement in serum creatinine, one patient had a remission of nephrotic range proteinuria, and one patient showed less endocapillary proliferation on a repeat kidney biopsy. However, two patients had a decline in renal function during treatment with eculizumab. Functional assays of the complement pathway may present a predictor of response to treatment. On the other hand Oosterveld et al. reported on the efficacy of eculizumab in 5 pediatric patients with DDD with either nephrotic syndrome or severe acute kidney injury who showed an activation of the alternative complement pathway. Treatment with eculizumab in these 5.9- to 13-year-old patients resulted in reduction of proteinuria and improvement of renal function in all 5 patients [46]. The variability of response to eculizumab in patients with DDD and C3GN suggests that pathophysiology of these diseases is more complex than pathophysiology of other complement-mediated diseases such as atypical hemolytic uremic syndrome. Age at presentation, duration of disease, and genetics of complement components are all likely to be important predictors of response to therapy with eculizumab, and these patients may be identifiable by a well-defined clinical, functional, and genetic characterization.

## 5. Rituximab for Recurrent MPGN after Kidney Transplantation

The recurrence rate of MPGN in complement-mediated disease or due to monoclonal gammopathy is higher than MPGN which is secondary to infection or autoimmune disease, and overall recurrence rates vary between 19 and 48 percent [47, 48]. A diagnosis of recurrent MPGN is strongly suspected in patients with a history of MPGN in their native kidneys who present after transplantation with new-onset proteinuria, hematuria, or renal failure. A kidney biopsy is the gold standard to establish the diagnosis of recurrent MPGN, although transplant glomerulopathy may be difficult to distinguish from MPGN. Electron microscopy helps to differentiate between those two pathologies, as does work-up for donor specific antibodies and C4d-positive staining of peritubular capillaries. Further, secondary causes including complement-mediated disease should be excluded as in MPGN of the native kidneys.

There is no evidence for an effective treatment of recurrent MPGN, although it appears reasonable to treat an underlying cause of MPGN. In a very recent paper Schrezenmeier et al. reported on the successful treatment of acute renal graft failure due to a recurrence of hepatitis C virus-associated MPGN with direct-acting antiviral agents (DAAs) daclatasvir and simeprevir [49]. Progressive disease, that is, presenting with nephrotic range proteinuria and worsening of renal function, can be treated with high doses of steroids, cyclophosphamide, and plasmapheresis, although outcome is uncertain [50, 51]. Few case reports suggest efficacy of rituximab treatment with or without plasmapheresis, but the level of evidence is very weak [52–54]. Rituximab is not an effective treatment of recurrent DDD after renal transplantation [55].

## 6. Summary and Conclusion

MPGN is a rare disease and current classification based on pathogenesis (i.e., histology, immunofluorescence, and analysis of the complement components) divides this disorder into immunoglobulin-associated MPGN (with or without secondary causes) and into complement-mediated disease which is termed C3GN and DDD. The optimal therapy for all varieties of MPGN is not known, and current KDIGO guidelines recommend treatment with steroids, cyclophosphamide with or without plasmapheresis only for subjects with progressive disease. Rituximab seems to be effective in immunoglobulin-associated MPGN, particularly in those cases associated with monoclonal gammopathy, chronic lymphocytic leukemia, and cryoglobulinemia with or without HCV, but severe adverse effect such as infections may limit its applicability. It remains to be shown how recently introduced DAAs will change also the course of HCV-associated renal diseases such as MPGN. On the other hand in complement-associated C3GN or DDD several case reports have shown no effect of rituximab on the course of these diseases.

In summary, the level of evidence for efficacy of any kind of treatment in a heterogenous and rare disease such as MPGN is very weak. Therefore, progress in determining the optimal therapy can only be achieved in large collaborative studies including patients with a clear diagnosis of a MPGN subtype in which treatment is tailored according to the subtypes using predefined study protocols.

## Figures and Tables

**Table 1:** *Studies of rituximab treatment in idiopathic MPGN*. RTX: rituximab, CreaCL: 24 h creatinine clearance, NA: not applicable or not reported, and CR and PR: complete and partial remission (as defined by the authors).

Authors	Study design	*n* (MPGN)	RTX protocol	Renal function	Proteinuria	Outcome
Sugiura et al. [37]	Prospective single-arm (*n* = 24)	1 (idiopathic)	1 × 375 mg/m^2^	Creatinine 0.51–1.95 mg/dl (whole cohort)	9.8 g/day	Proteinuria decreased from 9.8 → 1.8 g/day

Dillon et al. [36]	Prospective uncontrolled open-label (*n* = 6)	6 (4 idiopathic, 2 with cryoglobulinemia)	1000 mg on day 1 and on day 15	CreaCl 48 ± 13 ml/min/1.73 m^2^	3.9 ± 2.0 g/day	Proteinuria 2.1 ± 2.3 g/day
						CR in patients with cryoglobulinemia
						Stable renal function

Kong et al. [38]	Retrospective case review	2 (idiopathic)	1 × and 2 × 375 mg/m^2^	NA	NA	CR and PR

**Table 2:** *Reports on rituximab treatment in C3 glomerulopathy and dense-deposit disease*. RTX: rituximab, C3GN: C3 glomerulopathy, DDD: dense-deposit disease, MMF: mycophenolate mofetil, RAS: renin angiotensin system, C3Nef: C3 nephritic factor, TCC: terminal complement complex, CFH: complement factor H, CFI: complement factor I, MCP: membrane cofactor protein, and CR and PR: complete and partial remission (as defined by the authors).

Authors	Diagnosis	Follow-up	Other therapy	RTX protocol	Laboratory parameters	Outcome
Daina et al. [41]	DDD	5 months for RTX 48 months for eculizumab	Steroids Eculizumab RAS blockade	1 × 375 mg/m^2^	C3Nef positive C3 < 10% TCC high Genetics (CFH variants V62 and H402)	No effect of RTX on renal function or proteinuria PR with eculizumab

Rousset-Rouvière et al. [42]	DDD	21 months	Steroids MMF Eculizumab RAS blockade	2 × 375 mg/m^2^	C3 < 0.04 g/L C4 normal C3Nef positive Genetics: normal (CFH, CFI, MCP) Anti-CFH negative	Acute renal failure after RTX CR with eculizumab

Giaime et al. [40]	DDD	30 months	RAS blockade	4 × 700 mg weekly repeated after 18 months	Creatinine 235 *μ*mol/L Proteinuria 6 g/day C3 0.12 g/L (low) C4 normal C3Nef positive	Creatinine 200 *µ*mol/L Proteinuria < 0.5 g/L C3 remained low C3Nef remained positive

Payette et al. [43]	C3GN	72 months	Steroids MMF Eculizumab	4 × 375 mg/m^2^	Proteinuria 1–5.3 g/day C3Nef positive CFI mutation (I398L) anti-CFH positive low C3	No effect of RTX on proteinuria PR with eculizumab Normal renal function
